# Effects of firefighting on semen parameters: an exploratory study

**DOI:** 10.1530/RAF-20-0070

**Published:** 2021-03-11

**Authors:** Michelle Engelsman, Leisa-Maree L Toms, Xianyu Wang, Andrew P W Banks, Debbie Blake

**Affiliations:** 1 Fire and Rescue NSW, Greenacre, New South Wales, Australia; 2 School of Public Health and Social Work, Faculty of Health, Queensland University of Technology, Kelvin Grove, Australia; 3 QAEHS, Queensland Alliance for Environmental Health Sciences, The University of Queensland, Woolloongabba, Queensland, Australia; 4 Repromed, Remuera, Auckland, New Zealand

## Abstract

Firefighters are occupationally exposed to heat intensities and chemical concentrations that may affect fertility. Twenty firefighters participated in an exploratory study assessing fertility of firefighters via an online survey and semen analysis. Data analysis included consideration of demographic characteristics, reproductive history and occupational exposures. Overall, firefighter semen parameters were below World Health Organisation reference values designating fertility in men. Firefighters younger than 45 years had a higher incidence of abnormal semen parameters (42%) than those aged 45 years or greater (9%). Increased rank and higher levels of occupational and/or personal hygiene were associated with improved semen quality. Increased frequency of fire exposure was associated with a reduction in normal forms, volume, sperm concentration and total sperm count. Sperm clumping was greater than 10% in 26% of samples, suggesting reduced semen quality. This exploratory study provides novel data that support the hypothesis of an association between semen quality and firefighter’s occupational exposure to toxic environments.

Firefighters are occupationally exposed to heat intensities and chemicals known to affect fertility. As part of a wider study on firefighter exposure and reproduction, firefighters were recruited to contribute a semen sample to (1) evaluate semen parameters against fertility standards and other cohorts (2) assess demographic, exposure and reproductive history against semen analysis results, and (3) consider how occupational exposures may affect semen parameters. Following Human Ethics Approval through The University of Queensland, 20 firefighters contributed 23 semen samples at specified pathology centres. Semen samples were analysed in line with the World Health Organisation (WHO) methodology. Not all samples were analysed within 60 min due to unspecified pathology centre delays. Semen analysis included viscosity, liquefaction, agglutination, volume, sperm concentration, progressive motile, total motile, immotile, and normal forms.

Sample data were checked for completeness, consistency, accuracy and validity. Descriptive statistics were performed to summarise the data. Pearson’s correlations (two-tailed) were used to investigate the relationship between firefighter survey results and sample characteristics. Mann–Whitney *U-*tests were performed to determine statistical significance. Participants’ demographic and semen data, including WHO reference data for fertile men (WHO 2010), are shown in Table 1.

**Table 1:** Characteristics of participants in semen exploratory study, with WHO (2010) reference ranges presented. Data are presented as *n* or as mean ± s.d.

Characteristics	Values	WHO 2010
Total participants	20	
Age, years*	45 ± 10	
<45	11	
≥45	9	
Active duty (current fire exposure)	18	
Rank firefighter	18	
Rank station officer/captain	2	
Full-time firefighter	16	
Part-time firefighter	4	
Years in job*	20 ± 10	
Tobacco smoker	0	
Abstinence in days	3.8 ± 1.1	
Minutes to analysis	104 ± 66	
Successfully conceived at least 11 child	15	
Unable to conceive in 1 or more attempts	1	
Difficulty conceiving	6	
Unknown cause	4	
Abnormal semen parameters	1	
Hormone imbalance	1	
Underwent IVF in any instance	4	
Reported time to pregnancy	7	
≤12 months	5	
>12 months	2	
Experienced miscarriage(s)	3	
Negative pregnancy or birth outcomes^†^	7	
Semen volume		
5th%	0.6	1.5
50th%	2.0	3.7
95th%	5.0	6.8
Sperm concentration 10^6^/mL		
5th%	12	15
50th%	73	73
95th%	180	210
Total sperm count 10^6^/M		
5th%	33	39
50th%	150	260
95th%	450	800
Total motile %		
5th%	34	40
50th%	56	61
95th%	73	78
Progressive motility %		
5th%	16	32
50th%	46	55
95th%	71	72
Normal forms %		
5th%	3.1	4
50th%	9.0	15
95th%	21	44

*Age and duration of employment data were collected in 5-year increments (employment had one option of <1 year). To calculate the crude mean, the midpoint of each bracket was utilised. **These data are from firefighters self-reporting via the Stage 1 survey. ^†^Negative birth outcomes include miscarriage, still birth, pre-term birth, low birth weight, astigmatisms, attention deficit hyperactivity disorder (ADHD), club foot, dyspraxia, and asthma.

Across parameters, semen characteristics were grouped in relation to time to analysis (analysis within, or greater than, 60 min). No statistically significant differences were found based on time to analysis. Data were stratified by age (less than and greater than 45 years of age) based around research demonstrating statistically significant reductions in semen parameters with increasing age brackets above 45 years (Hellstrom *et al.* 2006). Younger participants (under 45 years of age) presented non-significant mean decreases in total motility (50% vs 61%), rapid progression (40% vs 53%) and morphology (8.7% vs 12%) when compared with those aged 45 years or greater. Frequency of exposure (less than or greater than weekly) was associated with non-significant mean decreases in morphology (7.8% vs 12%), volume (2.2 mL vs 2.8 mL), sperm concentration (80 M/mL vs 87 M/mL) and total sperm count (150 M/ejaculate vs 220 M/ejaculate).

Overall, firefighter semen parameters were below WHO reference values in numerous categories. Positive correlations (*P*  < 0.05) in semen quality were found across semen parameters with increased rank, with increased use of breathing apparatus across fire types, and with both increased showering post fire incidents and hand washing throughout the shift. Negative associations were detected for normal forms, volume, sperm concentration and total sperm count with increasing frequency of fire exposure. Sperm agglutination was >10% in 26% of samples.

This is the first investigation published on Australian firefighter sperm quality. Internationally, studies exist on firefighter reproductive history, with suggested links to toxic work (Petersen *et al.* 2019). There is, however, a scarcity of data on firefighter semen parameters. This exploratory study provides novel data that support the hypothesis that there is indeed an association between semen quality and firefighter’s occupational exposure to toxic environments. These results will add value to the design of a well-powered and targeted investigation aimed at maintaining and improving the health and well-being of firefighters, their families and offspring.

## Declaration of interest

There is no conflict of interest present within this research work. Although the lead researcher undertakes research while having a primary employment within a fire service, a comprehensive intellectual property, confidentiality and data sharing agreement has been signed between the lead researcher, employer and the University of Queensland, reducing any possibility of a conflict of interest.

## Funding

This work is funded predominantly by the Community Safety Directorate of Fire & Rescue NSW (FRNSW), Women and Firefighting Australasia (WAFA), and through substantial in-kind support by SafeWork NSW http://dx.doi.org/10.13039/501100012048 and TestSafe Laboratories.

## Author contribution statement

M E led study conception, the management of external relationships, participant recruitment, data analysis, and manuscript drafting, review and editing. L M T and X W were involved in study progression post inception, manuscript shaping, editing and review, and providing expert guidance. A P W B was involved in results analysis, manuscript shaping and editing. D B was involved in data analysis, manuscript shaping, editing and review, and providing expert guidance. All authors were involved in the discussion of results and consideration of limitations.
